# Asymptomatic Left Atrial Myxoma Treated With Minimally Invasive Surgical Approach

**DOI:** 10.7759/cureus.18432

**Published:** 2021-10-02

**Authors:** Tetyana Okan, Oleksandr Babliak, Kriti Agarwal, Yulia Kuzyk, Santh Prakash Lanka, Beshoy Iskander, Sanjeev Sharma, Satish Tadepalli, Richa Jaiswal, Akbar Hussain, Mohammed Y Rashid, Raja Chandra Chakinala

**Affiliations:** 1 Department of Diagnostic Imaging, The Metropolitan Andrey Sheptytsky Hospital, Lviv, UKR; 2 Department of Cardiac Surgery, Cardiac Surgery Center "Dobrobut", Kyiv, UKR; 3 Department of Internal Medicine, Hackensack Meridian Health Palisades Medical Center, North Bergen, USA; 4 Department of Pathological Anatomy and Forensic Medicine, Danylo Halytsky Lviv National Medical University, Lviv, UKR; 5 Department of General Surgery, Rangaraya Medical College, Kakinada, IND; 6 Department of Internal Medicine, Bon Secours Mercy Health- St. Elizabeth Youngstown Hospital (NEOMED), Youngstown, USA; 7 Department of Internal Medicine, Virginia Commonwealth University, Richmond, USA; 8 Department of Internal Medicine, Hackensack Meridian Health Ocean Medical Center, Brick, USA; 9 Department of Pathology, Medical University of South Carolina, Charleston, USA; 10 Department of Internal Medicine, Keystone Health System, Chambersburg, USA; 11 Department of General Surgery, University of Illinois -MGH, Chicago, USA; 12 Department of Internal Medicine, Geisinger Commonwealth School of Medicine, Scranton, USA; 13 Department of Internal Medicine, Guthrie Robert Packer Hospital, Sayre, USA

**Keywords:** transthoracic echocardiography (tte), minimally invasive surgery, minimally invasive cardiac surgery, trans thoracic echocardiography, heart neoplasm, benign cardiac tumor, asymptomatic myxoma, left atrial myxoma

## Abstract

Myxomas, being the most common primary benign tumor among all cardiac tumors, occur rarely with a very low incidence rate. Myxomas can cause various clinical manifestations or can be diagnosed incidentally. Some patients with cardiac myxomas are completely asymptomatic. Cardiac myxomas cause life-threatening complications, thus early diagnosis is imperative. We are reporting a case of atrial myxoma in a 38-year-old female without any significant past medical history, who came to our clinic for cardiology evaluation prior to plastic surgery. The elliptical mobile mass, located in the left atrium with its attachment to the interatrial septum, was diagnosed by transthoracic echocardiography. The patient was referred for surgery and a minimally invasive surgical approach was chosen. A histological report confirmed the diagnosis of myxoma. The patient recovered well. Three years of follow-up did not reveal any signs of recurrence of the tumor. We are also analyzing 20 previously published cases of asymptomatic myxomas and myxomas treated with a minimally invasive surgical approach, reported in the PubMed database for the last 20 years (2001-2021) in the adult patient population (age 19 and over). The aim of this study is to highlight the asymptomatic presentation of cardiac myxomas and to underline the advantages of a minimally invasive surgical approach. In summary, asymptomatic cardiac myxomas are rare incidental findings. Attention to subtle symptoms during a physical exam and scrupulous history-taking can provide a clue to this diagnosis. Early diagnosis of cardiac myxomas is crucial to prevent life-threatening complications. Minimally invasive surgery is a promising alternative approach to standard open-heart surgery for treating cardiac myxomas, providing faster recovery and higher patient satisfaction with surgical care.

## Introduction

Cardiac tumors are differentiated into primary and secondary. The prevalence of primary heart tumors is 0.001-0.03%, as reported in the autopsy series [1]. Seventy-five percent of all primary cardiac tumors are benign in origin. Myxoma is the most common benign tumor accounting for 50-70% of all primary benign tumors while angiosarcoma is the most common malignant tumor accounting for 30% of all malignant cases, followed by rhabdomyosarcoma with its frequency of 20% [2]. Only one-fifth of all myxomas are totally asymptomatic. Most cases of heart myxomas are sporadic. Multiple tumors occur rarely. However, in the case of Carney complex, a heritable autosomal dominant disorder, multiple myxomas occur in 50% of cases with a more frequent location in the ventricle (13%) [3]. The female-to-male ratio is in the range from 2:1 to 3:1 [3]. Myxoma, originating from undifferentiated and totipotent mesenchymal stem cells, produces a vascular endothelial growth factor that is responsible for the early growth of myxoma due to induction of angiogenesis [1,4]. Some characteristics of myxoma, such as locally invasive growth, its extension outside the heart, cases of recurrence, and reported distant metastasis (brain, sternum, vertebrae, scapula, pelvis) suggest its malignant features [4].

## Case presentation

**Figure 1:**
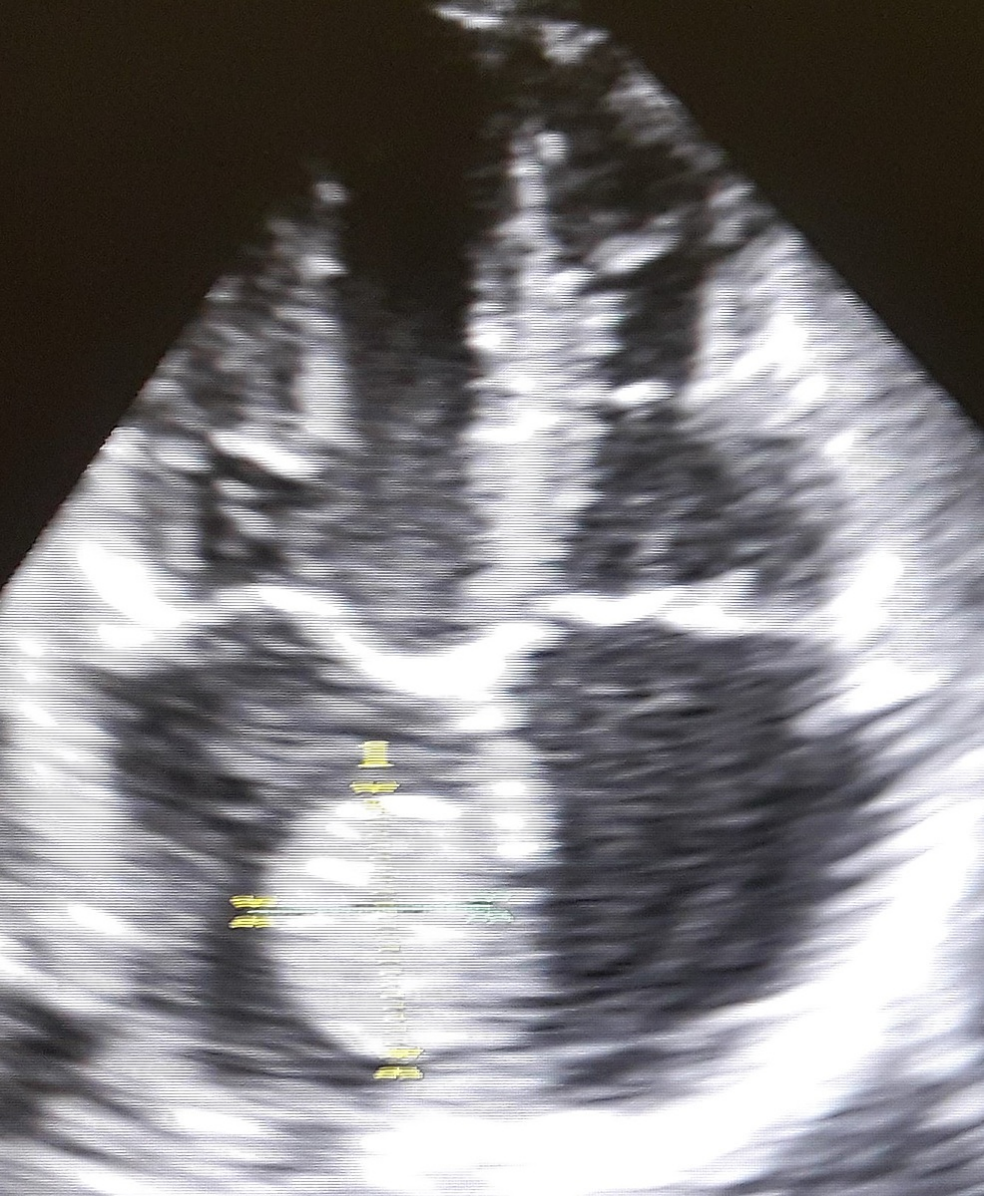
Myxoma in the left atrium. Transthoracic echocardiographic study, apical 4 chamber view. Left atrial mobile homogeneous hyperechoic mass, attached to interatrial septum.

A 38-year-old white asymptomatic female without any significant past medical history or risk factors came to our clinic for cardiology consultation prior to cosmetic facial surgery. The patient, who recently returned from a mountain climbing trip in the Himalayas, was in good physical shape. On a physical examination a holosystolic murmur, best heard at the left lower sternal border, was noticed. The patient was sent for transthoracic echocardiography (TTE), which showed elliptical, mobile homogeneous hyperechoic mass, 28x24 mm, with smooth surface, located in the left atrium (Figure 1).

The mass did not prolapse through the mitral orifice during diastole, did not obstruct diastolic filling of the left ventricle, was attached to the interatrial septum, although the peduncle was not clearly visualized (Video 1). The left atrial diameter was on the upper limit of 3.8 cm. Color Doppler did not reveal increased velocity of blood flow across the mitral valve. Moderate tricuspid insufficiency was noticed. A diagnosis of a left atrial tumor was made. The findings were discussed with the patient and she was referred to a specialized Cardiac Surgery Center for surgical intervention. A minimally invasive approach was chosen. 

**Video 1:** Transthoracic echocardiographic study. Apical five-chamber view. Left atrial mobile myxoma.

The patient underwent a right lateral mini-thoracotomy under general anesthesia. Peripheral cardiopulmonary bypass using right femoral artery and vein and right internal jugular vein was established. Induced ventricular fibrillation was used. Left and right atria were opened. The tumor was excised with a part of an interatrial septum (Video 2), which was closed using autopericardial patch. Heart chambers were closed, cardiopulmonary bypass was discontinued and the operation was finished in the usual manner. The total operation time was 220 minutes. The postoperative period was uncomplicated. The total hospital stay was four days. 

**Video 2:** Operative intervention showing removal of atrial myxoma.

**Figure 2:**
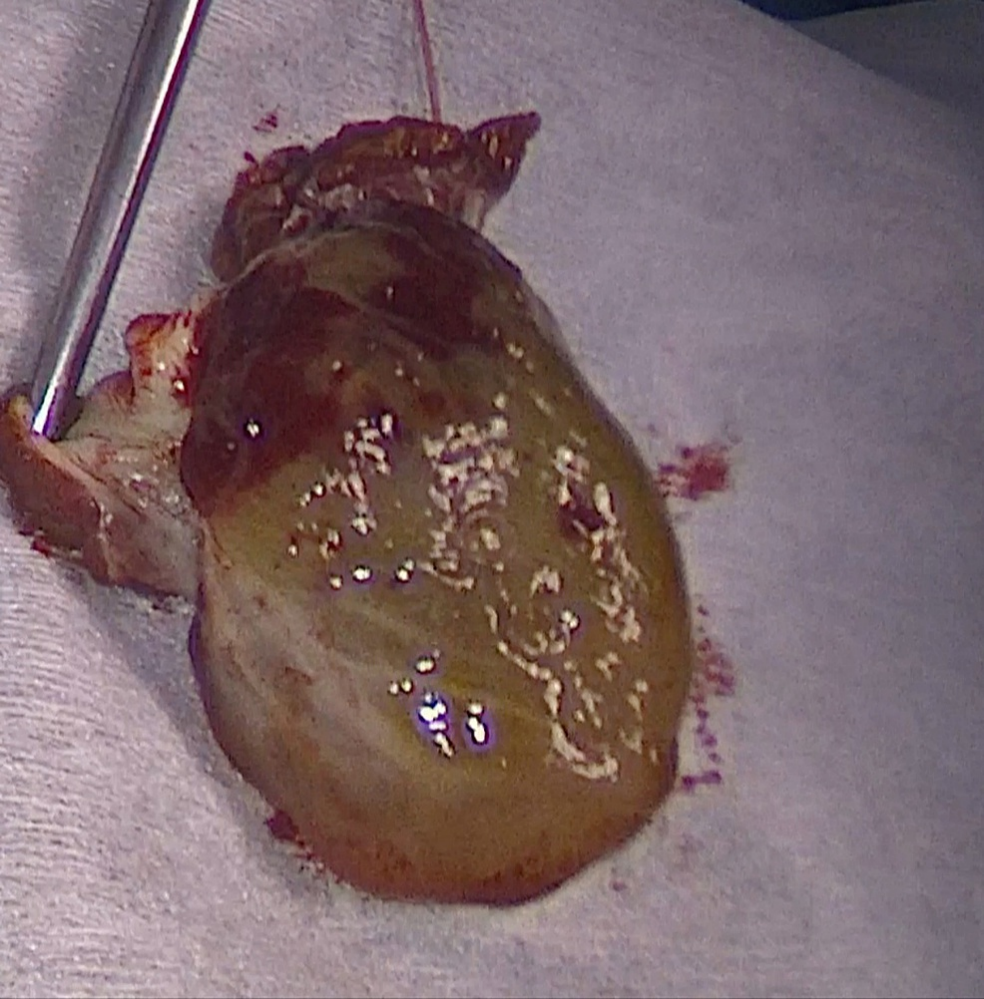
Gross-examination of operation material: oval-shaped tumor consisting of gelatinous tan-white tissue with friable surface.

The histopathological report confirmed a diagnosis of cardiac myxoma. Macroscopically, the specimen consisted of a small fragment of the left atrial wall and the oval-shaped tumor, presented by gelatinous tan-white tissue with the friable surface (Figure 2).

**Figure 3:**
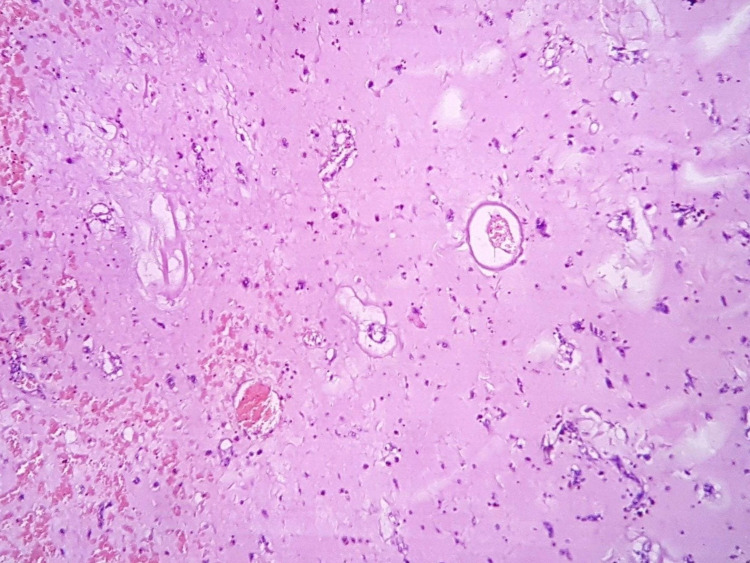
Typical structure of myxoma in the form of clusters of multiple stellate and spindle cells among myxomatous stroma. Hematoxylin and eosin stain ×200 (original magnifications).

Microscopically, hematoxylin and eosin-stained sections showed a neoplastic lesion with two components. The first, a cellular component, consisting of stellate and spindle cells with scant eosinophilic cytoplasm, round to oval nuclei (some multinucleated), and mild nuclear polymorphism (Figure 3). No significant cytologic atypia, necrosis, or stromal desmoplasia was observed.

**Figure 4:**
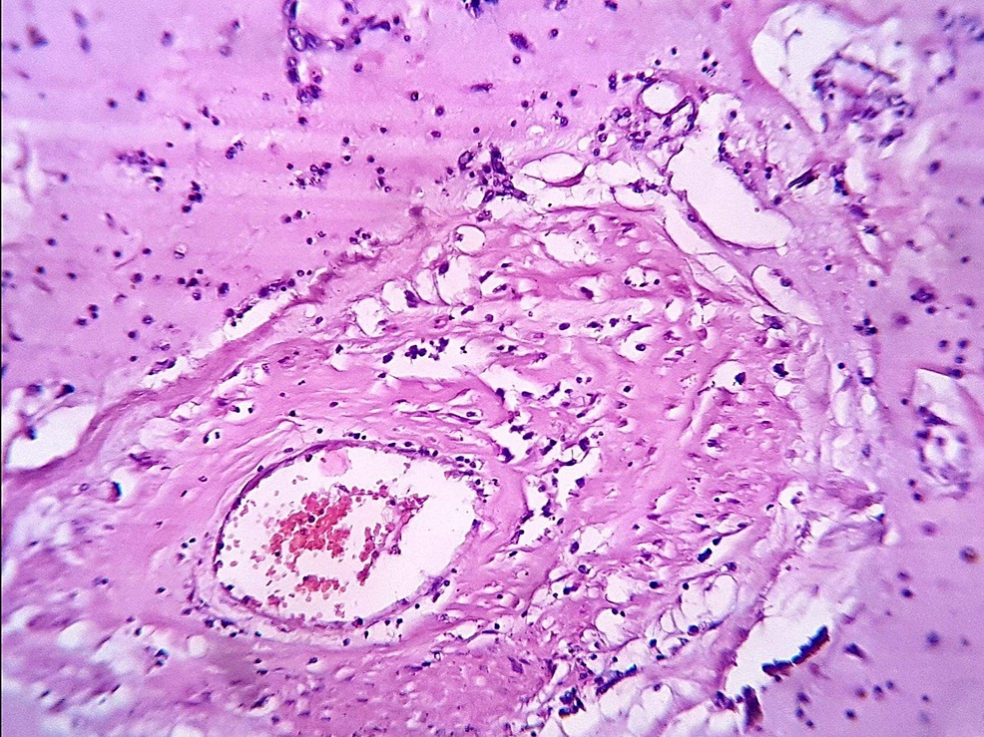
Extracellular component of the tumor: a large vessel in the tumor, represented only by myxomal cells, there are accumulations of hemosiderin granules around the vessel. Hematoxylin and eosin stain ×200 (original magnifications).

The second, extracellular component, was represented by an edematous eosinophilic stroma with foci of hyalinosis, numerous vessels, surrounded by accumulations of hemosiderin granules. Small vessels of the tumor had capillary type structure and resembled vascular slits and wide tubular formation. Large vessels consisted of only several layers of myxomal cells. The basement membrane, smooth muscle cells, and adventitia were absent (Figure 4). It should be noted that the structural features of the vessels in the myxoma cause their slight vulnerability during traction in the turbulent blood-contracting chambers of the heart. This was confirmed by multiple hemorrhages in the tumor stroma of different ages.

The postoperative period was uncomplicated. The patient was followed up annually for three years with no signs of recurrence of the tumor.

## Discussion

We present a rare case of a completely asymptomatic patient with left atrial myxoma, which was incidentally revealed during preoperative cardiac evaluation prior to plastic facial surgery. Twenty previously published cases of asymptomatic myxomas and cases of myxomas treated with a minimally invasive surgical approach, reported in the PubMed database for the last 20 years (2001-2021) in the adult patient population (age 19 and over) were analyzed. We summarized information to present a comprehensive review, including information about the first authors of the studies, year of publication, demographics, initial presentation, imaging tests, size of myxoma, localization, and site of its attachment, type of surgery, and postoperative follow-up.

Asymptomatic myxomas, described in previously published case reports, were found either during routine check-ups or due to work-ups for other medical conditions, such as hypertension, chronic liver disease, bronchiectasis disease, or due to necessity to be admitted to the hospital because of cancer, bronchocele surgery, elective gastric banding. One of the patients underwent TTE screening as her daughter has been diagnosed with hypertrophic cardiomyopathy (HCM) and was found both HCM and myxoma [5]. One of the patients has been revealed to have myxoma two months after radiofrequency ablation (RFA) procedure and five months post-procedure it increased in size, thus it was removed [6]. One of the patients with a history of myxoma removal has been diagnosed with a recurrent tumor during control TTE [7]. In contrast to our case, commonly about 80 % of myxomas are symptomatic with such typical clinical manifestations, as cardiac symptoms (67%), including signs of valvular obstruction (dyspnea, orthopnea or platypnea, paroxysmal nocturnal dyspnea, chest pain) and direct invasion of the myocardium (decreased contractility, arrhythmias, and heart blocks); embolic (29%), mostly left-sided; and systemic (34%) symptoms (fever, anorexia, weight loss, fatigue, arthralgia, myalgia, and Raynaud’s phenomenon) [3,8]. Symptoms often depend on a change in body position.

Myxoma in the reported case was located in the left atrium, attached to the interatrial septum. About 75% (60-88%) of myxomas occur in the left atrium (the area of the fossa ovalis is the usual site of attachment), 15-20% (4-28%) occur in the right atrium [9,10]. Atypical locations, described in the literature, include: posterior or anterior left atrium wall, atrial appendage ridge, arising from the ostium of the coronary sinus, left ventricle (3-8%), right ventricle (3-6%), mitral valve (6,1 %), including chordae of the mitral valve, anterior or posterior mitral leaflets or mitral annulus, biatrial location (<2.5%) with extending tumor through the foramen ovale into the contralateral atrium, the aortic valve, biventricular tumors [9,10]. Patients having cardiac myxoma in a cardiac chamber other than the left atrium, presenting at a younger age, having multiple tumors in multifocal locations are at higher risk to be diagnosed with Carney complex, a familial disorder with spotty pigmentation of the skin and endocrinopathy.

Tumors vary widely in size, ranging from 1 to 15 cm in diameter and weighing between 15 g and 180 g [3]. Myxomas grow rather fast. The calculated growth rate shows an average growth rate of 0,49 cm/month [11]. Huge myxomas undergo variable degrees of regressive changes with evidence of areas with hemorrhage, necrosis, calcification, and cyst formation. Friable, villous, irregular tumors increase the risk of systemic embolism. However, even small and nonmobile cardiac myxomas with a round regular shape may cause recurrent cerebral infarction.

Transthoracic echocardiography is a simple and non-invasive screening method of diagnosing heart masses, detecting 95% of cardiac masses [12]. However, two-dimensional echocardiography has its limitations, such as tiny tumors may be missed, planar imaging is not always representative for asymmetric structures, ultrasound artifacts may lead to an incorrect diagnosis of nonexisting intracardiac mass. Transesophageal echocardiography (TEE), real-time three-dimensional echocardiography, magnetic resonance imaging, and computed tomography provide additional important information, such as tissue characteristics, precise location, its extent, and thus resectability.

The standard approach in treating cardiac myxoma is the median sternotomy. Two-thirds of patients in the previously published cases underwent standard surgery. However, standard median sternotomy is associated with unsatisfied cosmetic outcomes and risk of sternal infection. In our case, the patient underwent a less frequently used minimally invasive surgery and it was successful without any postoperative complications. The patient recovered well, staying in the hospital only for four days, and was satisfied with the surgical care, expedited wound healing, and small size of the cosmetic scar. Right anterolateral minithoracotomy with incision of 4 to 6 cm is gradually applied in the surgery of cardiac tumors and is a good alternative technique, compared to standard open-heart surgery, for treating cardiac myxomas. It is associated with reduced trauma and pain, low complication rates, significantly fewer arrhythmia events, a shorter intensive care unit, and hospital stay: median duration of hospital stay in the minimally invasive group is 10.4 ± 1.5 days vs sternotomy group 17.5 ± 5.6 (p = 0.004), according to Sawaki S. et al. [13,14]. Patients with minithoracotomy, in contrast to sternotomy, have less postoperative chest drainage (536 vs 773 ml, P < 0.01), less transfusion rate (5.9% vs 33.3%, P = 0.033) and are more satisfied by the cosmetic healing of the wound, according to Luo C. et al [15]. Long-term outcomes following complete resection of the tumor are excellent, a postoperative mortality rate is 0-3%. Recurrence of cardiac myxoma was observed in about 3% in sporadic cases, and 20% in Carney complex. The possible causes of recurrence include incomplete resection of the tumor, implantation from the original tumor, unrecognized multicentric origin, or new growth of tumor [4]. Recurrences are characterized by faster and more infiltrative growth compared to original tumors [4]. Serum interleukin-6 levels may be raised, thus it can be used as a marker of recurrence [4]. Other immunological markers of myxoma are erythrocyte sedimentation rate (ESR), C-reactive protein (CRP), interleukin-2R (Il-2R), and intracellular adhesion molecule (ICAM) [16], however, these markers are non-specific.

Detailed information is presented in the table (Table 1).

**Table 1:** Previously published cases of asymptomatic myxomas and myxomas treated with minimally invasive surgery, their demographics, initial presentation, imaging tests, size of myxoma, localization and site of its attachment, type of surgery, and postoperative follow-up.

	Reference, year	Pt. age (yr), sex	Initial presentation	Imagine test	Localization, size (mm)	Type of surgery	Recovery and follow-up
1	Lamparter S, et al, 2004 [17]	70 F	asymptomatic, admitted to hospital with a diagnosis of hepatic metastases originating from colorectal cancer	chest CT, TTE	LA, attached to the posterior left atrial wall, prolapsing into the mid-left ventricular cavity; 100 x 30	surgical excision	uneventful recovery
2	Panagiotou M, et al, 2008 [18]	58 M	asymptomatic, incidental finding during a work-up for hemoptysis due to bronchectasis	chest CT, TEE	LA, attached to interatrial septum by a stalk; multi-lobulated, with excessive osteoid content; 120 x 100	surgical excision	uneventful recovery, except the need for temporary external pacing; f/u for 2 years: no recurrence
3	Ozer N, et al, 2009 [19]	58 F	asymptomatic, h/o breast ductal adenocarcinoma, referred for the evaluation of the potential cardiotoxic side effects of anthracycline-based chemotherapy	TTE, TEE	RA, highly mobile, cystic mass on a broad base, attached to the lower dorsal free wall, interatrial septum and upper border of the inferior vena cava; 20 x 25	surgical excision	uneventful recovery
4	Charokopos NA, et al, 2009 [8]	30 M	asymptomatic, routine cardiologic workup due to hypertension	TTE, TEE	LV, attached to the ventricular surface of the anterior mitral leaflet; 17 x 20	surgical excision	uncomplicated recovery; f/u for 6 months
5	Charokopos NA, et al, 2009 [8]	65 F	asymptomatic, admitted in order to have surgery for a bronchocele	TTE, TEE	RA, attached to the atrial wall by a narrow pedicle, protruding through the tricuspid valve into the right ventricle; 65 x 55 x 45	surgical excision	uncomplicated recovery; f/u for 10 months
6.	Modi P, et al, 2009 [20]	65 M	asymptomatic, undergoing echocardiographic work-up for a cardiac murmur	TTE	at the junction of the mid and apical lateral segments of the left ventricle; 12 x 19	right anterior minithoracotomy	uneventful recovery; f/u for 2 month
7.	Darwazah AK, et al , 2011 [21]	26 F	asymptomatic, h/o morbid obesity, admitted for elective gastric banding	TEE, MRI (TTE did not reveal a tumor, but showed a small atrial septal defect)	RA, arising from the inferior vena cava; 10 x 9	surgical excision	uneventful recovery; f/u for 2 years: no recurrence of tumor
8.	Abdou M, et al, 2013 [5]	71 F	asymptomatic, routine TTE, as her daughter has been diagnosed with hypertrophic cardiomyopathy (HCM)	TTE screening, confirmed with TEE	LA, arrising from the interatrial septum; 12 x 22 The patient also has been diagnosed with HCM (IVS - 27 mm)	standard surgical excision and ICD placement	uneventful recovery
9.	Rubio Alvarez J, et al, 2013 [6]	60 M	asymptomatic, 2 month after radiofrequency ablation (RFA) procedure, referred for MRI to evaluate the right ventricular anatomy	MRI (2 months after RFA), TTE (5 months after RFA)	LA, attached to interatrial septum; 10 x 10; in 3 month: 20 x 20	standard surgical excision	uneventful recovery; f/u for 3 years: no recurrence
10.	Abad C, et al, 2014 [22]	69 F	asymptomatic, hospitalized because of chronic liver disease	TTE, TEE	LV, with a stalk attached to the left ventricular endocardium; 13 x 23	surgical excision	f/u for 3 month
11.	Espinola-Zavaleta N, et al, 2014 [23]	55 F	asymptomatic, h/o colon adenocarcinoma	routine chest CT, TEE 3D	LA, attached to the middle portion of the interatrial septum by a small pedicle; 24 x 21 x 14	surgical excision	uneventful recovery; f/u for 2 years: no recurrence
12.	Espinola-Zavaleta N, et al, 2014 [23]	66 F	asymptomatic, h/o chronic intermittent diarrhea	thoraco-abdominal CT, TEE 3D	LA, attached to the roof of the left atrium by a short, thick pedicle; dystrophic ossification; 27 x 22	surgical excision	uneventful recovery; f/u for 1,5 years: no recurrence
13.	Strecker T, at al, 2014 [24]	62 F	asymptomatic, routine medical check-up	TTE	RA, attached to the lateral wall of the RA; 41 x 46	median sternotomy	post‐operative course was uneventful
14.	Łebek-Szatańska AM, et al, 2016 [7]	75 F	asymptomatic, h/o LA myxoma (33 x 57), removed 1.5 years ago	control TTE	recurrent myxoma in LA, attached to the interatrial septum; 22 x 37	no surgery	f/u for 10 years, repeatedly refused reoperation
15.	Tarui T, et al, 2016 [25]	68 M	asymptomatic	TTE, chest CT	LA, attached to interatrial septum; 19×15×17	robot-assisted using da Vinci S Surgical System	uneventful recovery; f/u for 6 months: no recurrence
16.	Uchida N, et al, 2018 [26]	75 F	asymptomatic, h/o intraductal papillary mucinous neoplasm, came for a regular follow-up	endoscopic ultrasound of mediastinum, CT with contrast	LA, attached to interatrial septum; 15 x 9	surgical excision	uneventful recovery
17.	Misawa Y, et al, 2002 [27]	74 M	paroxysmal atrial fibrillation	TTE, chest CT	superior wall of the left atrium; 28 × 25	endoscope-assisted superior septal approach	uneventful recovery
18.	Olsthoorn J, et al, 2018 [28]	49 F	progressive dyspnea and palpitations	TTE	LA, attached to interatrial septum; 45x 60	minimally invasive approach	uneventful recovery; f/u for 3 month
19.	Szerszyńska A, et al, 2019 [29]	24 M	recurrent right-sided pneumonia, chronic PE	chest CT angio, TEE	RV, attached to the RV apex; 26 x 12	right mini-thoracotomy	1 month postop: suspected IE or thrombi in the RA, resolved in 6 days of appropriate treatment; f/u for 1 year: no recurrence
20.	Kim CH, et al, 2020 [30]	24 M	recurrent fever and stroke, misdiagnosed as infective endocarditis; h/o Cushing’s syndrome, resected adrenal adenoma. Carney complex (PRKAR1A mutation)	TTE	LA, attached to the interatrial septum; 70	right mini-thoracotomy	uncomplicated recovery

Limitations of the study: a small sample size, we were limited to full-text English language case reports only, we did not include population under age 19 as well as articles not containing enough information about variables we were gathering.

## Conclusions

Asymptomatic cardiac myxomas are rare primary cardiac tumors. However, they should be included in the differential diagnosis of non-specific cardiothoracic symptoms, as myxomas cause life-threatening complications. Noticing mild signs and symptoms during a physical examination and taking a detailed history is imperative to diagnose this tumor. The minimally invasive surgical approach has multiple advantages comparing to standard open-heart surgery: lower complications rates, lower risk of infection, less blood loss, fewer arrhythmia events, shorter intensive care unit stay and hospital stay, higher patient satisfaction with cosmetic healing of the wound, scar appearance, and surgical care. Minimally invasive surgery is a promising technique for treating cardiac myxomas. Further studies are needed to better estimate the advantages of the minimally invasive cardiac approach.
